# High-Permittivity Carbonized Kapok Fiber/Waterborne Polyurethane Composite Films as Frequency-Selective Filtering Layers for Thin-Profile Multiband Microwave Absorption

**DOI:** 10.3390/molecules31213985

**Published:** 2026-11-05

**Authors:** Aiqiong Wang, Yichen Lin, Zhenrong Zheng, Xiaoming Zhao

**Affiliations:** 1 School of Art and Media, Quanzhou Vocational and Technical University, Quanzhou 362268, China; 2 School of Textile Science and Engineering, Tiangong University, Tianjin 300387, China; 3 Ministry of Education Key Laboratory for Advanced Textile Composite Materials, Tiangong University, Tianjin 300387, China

**Keywords:** carbonized kapok fiber, waterborne polyurethane, wave-filtering layer, high permittivity, thin-profile multiband microwave absorption

## Abstract

Thin-profile broadband microwave absorbers must reconcile impedance matching and strong attenuation, two requirements that usually pull in opposite directions. Here, hollow carbonized kapok fibers (CKFs) obtained at 1000 °C were compounded with waterborne polyurethane (WPU) at a mass ratio of 8:2 and consolidated by hot pressing. The film reached a dielectric real part of about 325 and behaved as a frequency-selective layer: it transmitted low-frequency microwaves while reflecting high-frequency ones, governed by the frequency-dependent permittivity, the skin effect, a narrow resonance window, mesopore scattering, and the impedance mismatch with free space. Inserting a 0.62 mm CKF/WPU interlayer between a high-frequency surface layer and a low-frequency bottom layer broadened the effective absorption bandwidth from 4.71 to 9.27 GHz (windows at 3.33–9.30 and 11.6–14.9 GHz), with a minimum reflection loss of −54.8 dB at a total thickness of only 1.53–1.62 mm, covering the whole C band and parts of the S and X bands. This provides a low-cost route to thin, flexible multiband microwave absorbers through a biomass-derived filtering interlayer.

## 1. Introduction

The rapid expansion of wireless communications, radar systems, and consumer electronics has turned electromagnetic interference into a recurring problem, both on modern battlefields and in daily life [1,2]. Microwave-absorbing materials, which convert incident electromagnetic energy into heat rather than reflecting it, have therefore attracted steady attention over the past decades [3,4]. An ideal absorber must satisfy two competing requirements at once: the impedance should be close enough to free space that most of the incident wave enters the material, while the attenuation constant should be large enough that the entered energy is dissipated rapidly through dielectric or magnetic loss [5]. Reconciling the two is not trivial, and combining strong absorption, broad bandwidth, and small thickness in a single material remains an open challenge [6].

The C band (4–8 GHz), together with the adjacent S (2–4 GHz) and X (8–12 GHz) bands, is heavily used in satellite links, base stations, and radar [7,8]. Absorption in this region is intrinsically difficult [9,10]. The long wavelengths demand either a thick layer that meets the quarter-wavelength condition or a high loading of magnetic fillers, both of which conflict with the lightweight, thin-profile, and flexible requirements of wearable and airborne applications [11].

Multilayer design is one of the most effective ways around this trade-off [8]. By assigning a distinct electromagnetic function to each layer, a broadband response can be achieved without increasing the total thickness [12,13]. A particularly useful building block is a wave-filtering layer that transmits low-frequency microwaves and reflects high-frequency ones, placed between a high-frequency surface layer and a low-frequency bottom layer [14]. The whole structure then extends absorption across multiple frequency windows, from the low-frequency side to the high-frequency side [15,16]. The key is a filtering material with a high, strongly frequency-dependent permittivity: its mismatch with free space is large enough to reflect short-wavelength waves, yet weak enough for long-wavelength waves to enter and reach the bottom layer [17,18].

Carbonaceous materials are natural candidates for such a layer because of their tunable conductivity, low density, and abundant interfacial polarization [19,20]. Among biomass precursors, kapok fiber is especially attractive: it is a natural hollow tube with a thin wall and an extremely low density, and it can be converted directly into hollow carbon microtubes by pyrolysis in an inert atmosphere [21]. The hollow lumen and the nanopores generated during carbonization provide internal reflection interfaces and scattering centers that help regulate wave propagation [22]. Waterborne polyurethane (WPU), an environmentally friendly and flexible binder, is a suitable matrix for turning these microtubes into a free-standing film [23]. Recent reports on fiber-derived flexible absorbing structures [24] and on compositionally complex systems with strong electromagnetic responses [25] further confirm the potential of this design strategy [26].

In this work, carbonized kapok fibers (CKFs) obtained at 600–1200 °C were compounded with WPU at different mass ratios and consolidated by hot pressing. The CKF prepared at 1000 °C, combined with WPU at a mass ratio of 8:2, gave the highest permittivity and the most pronounced frequency-selective behavior. Inserting a 0.62 mm CKF/WPU interlayer into a sandwich structure widened the effective absorption bandwidth from 4.71 to 9.27 GHz at a total thickness of only 1.53–1.62 mm, covering the whole C band and parts of the S and X bands. The result offers a low-cost, scalable route from natural hollow fibers to thin, flexible, broadband absorbing composites. Moreover, the resulting flexible CKF/WPU film conforms to curved surfaces, a feature relevant to aerospace skins, drone wings, and wearable personal-protection garments, where thin, light, and bendable absorbers are required.

## 2. Results and Discussion

### 2.1. Morphology, Pore Structure, and Physicochemical Characterization

**Figure 1:**
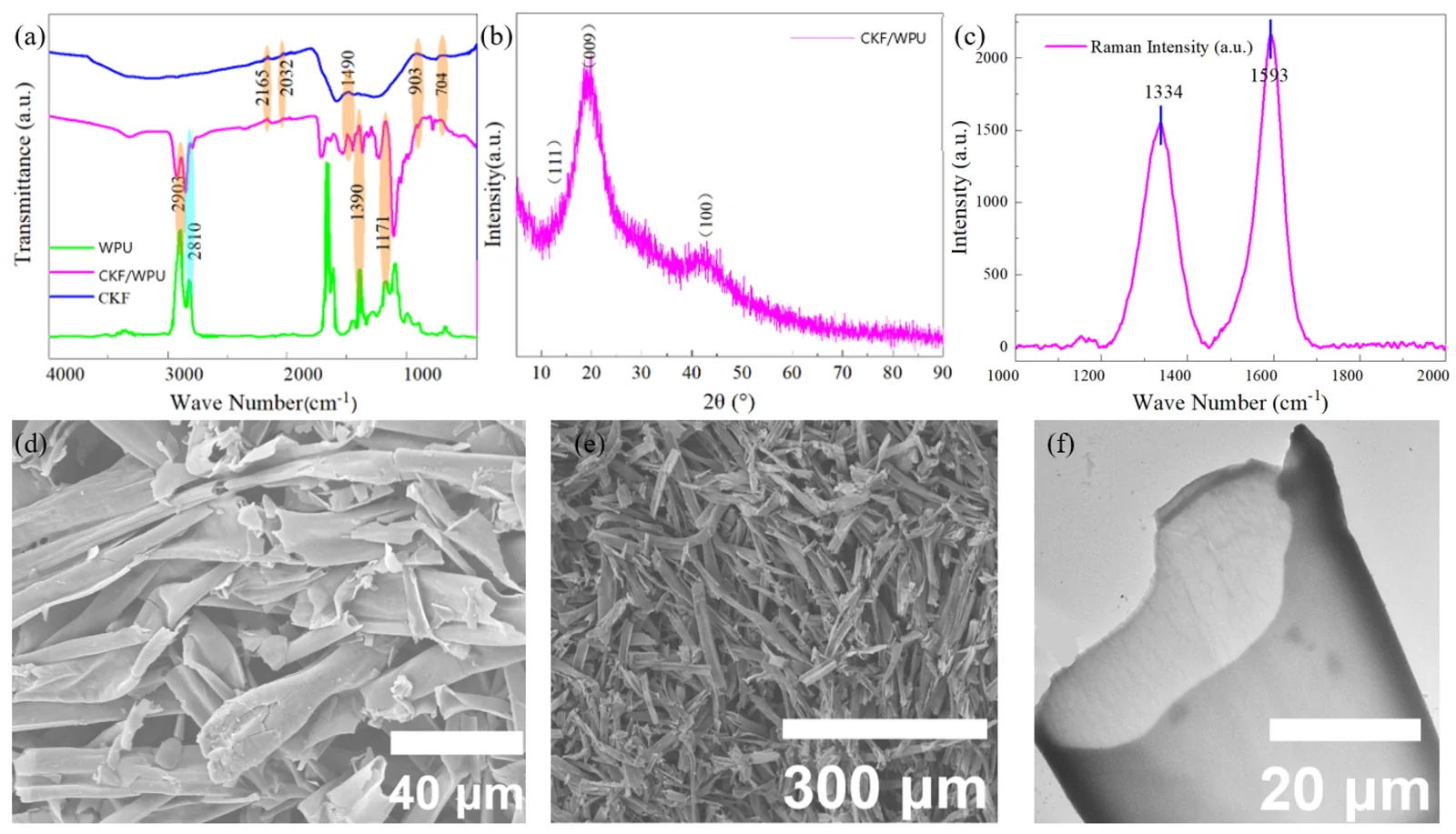
(**a**) FTIR spectra of CKF, WPU, and CKF/WPU. (**b**) XRD pattern of CKF/WPU. (**c**) Raman spectrum of CKF-3 with the D band at 1334 cm^−1^ and the G band at 1593 cm^−1^ (ID/IG ≈ 0.73). Three measurements at different positions give nearly identical spectra. (**d**,**e**) SEM images of CKF and CKF/WPU. (**f**) TEM of CKF.

The FTIR spectra of CKF, WPU, and CKF/WPU are presented in Figure 1a. The CKF/WPU spectrum shares peaks with CKF and WPU at 704, 903, 1409, 2032, and 2165 cm^−1^, together with additional common peaks at 1171, 1390, 2810, and 2903 cm^−1^. The retention of these characteristic peaks indicates that the functional groups of both components survive in the composite film [27]. The XRD pattern of CKF/WPU is shown in Figure 1b. According to the standard card PDF #49-1720, the peak at 2θ = 12.75° corresponds to the (111) plane of a fullerene-type structure. According to PDF #50-0926, the peaks at 2θ = 29.11° and 42.63° correspond to the (009) and (100) planes of graphitic carbon, respectively. The XRD results show that CKF contains not only the typical structural features of common carbon materials but also fullerene-type features, indicating that both fullerene and graphitic carbon structures are integrated in CKF/WPU [28].

The Raman spectrum of CKF-3 is shown in Figure 1c. Two characteristic bands appear: the D band at 1334 cm^−1^, assigned to the disorder-induced breathing mode of sp^2^ carbon, and the G band at 1593 cm^−1^, assigned to the in-plane vibration of graphitic carbon. The peak-height ratio ID/IG is about 0.73, which points to a moderate graphitization degree with a measurable number of structural defects, as shown in Table S1. The graphitic domains carry the conduction current, and the defects act as polarization centers, so the two together support the high permittivity discussed later [29]. Three measurements taken at different positions (1-1 to 1-3) give nearly identical spectra, so the fibers are uniform on the scale probed by the laser.

The SEM images of CKF and CKF/WPU are shown in Figure 1d,e. Statistical analysis indicates that the average diameter and length of the CKF within the CKF/WPU film are 10.77 and 314.6 μm, giving an aspect ratio of about 29.2 (Figure S1a,b), a moderate value for carbonized kapok fibers. After high-temperature oxygen-isolated annealing, the hollow cavity of the kapok fiber collapses and the distance between the fiber walls decreases, which favors the formation of a thin three-dimensional conductive network and an electromagnetic wave conduction pathway [30]. The TEM image in Figure 1f shows the fractured cross-section and the thin fiber wall of CKF. Statistical analysis (Figure S1c) gives a fiber wall thickness of only 0.88 μm.

### 2.2. Electromagnetic Properties and Compositional Optimization

**Figure 2:**
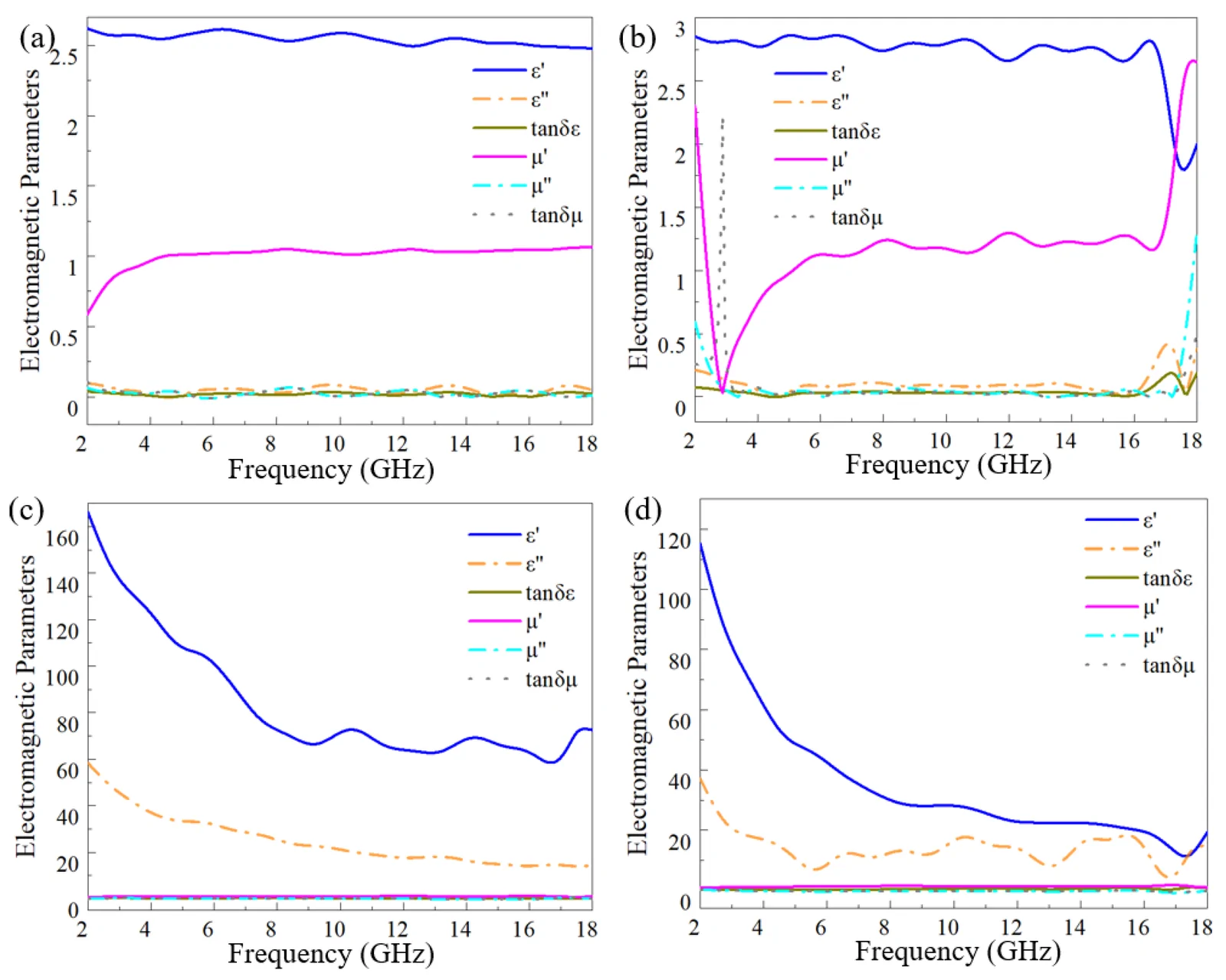
Electromagnetic parameters of (**a**–**d**) CKF-1 to CKF-4 measured in the range of 2–18 GHz.

**Figure 3:**
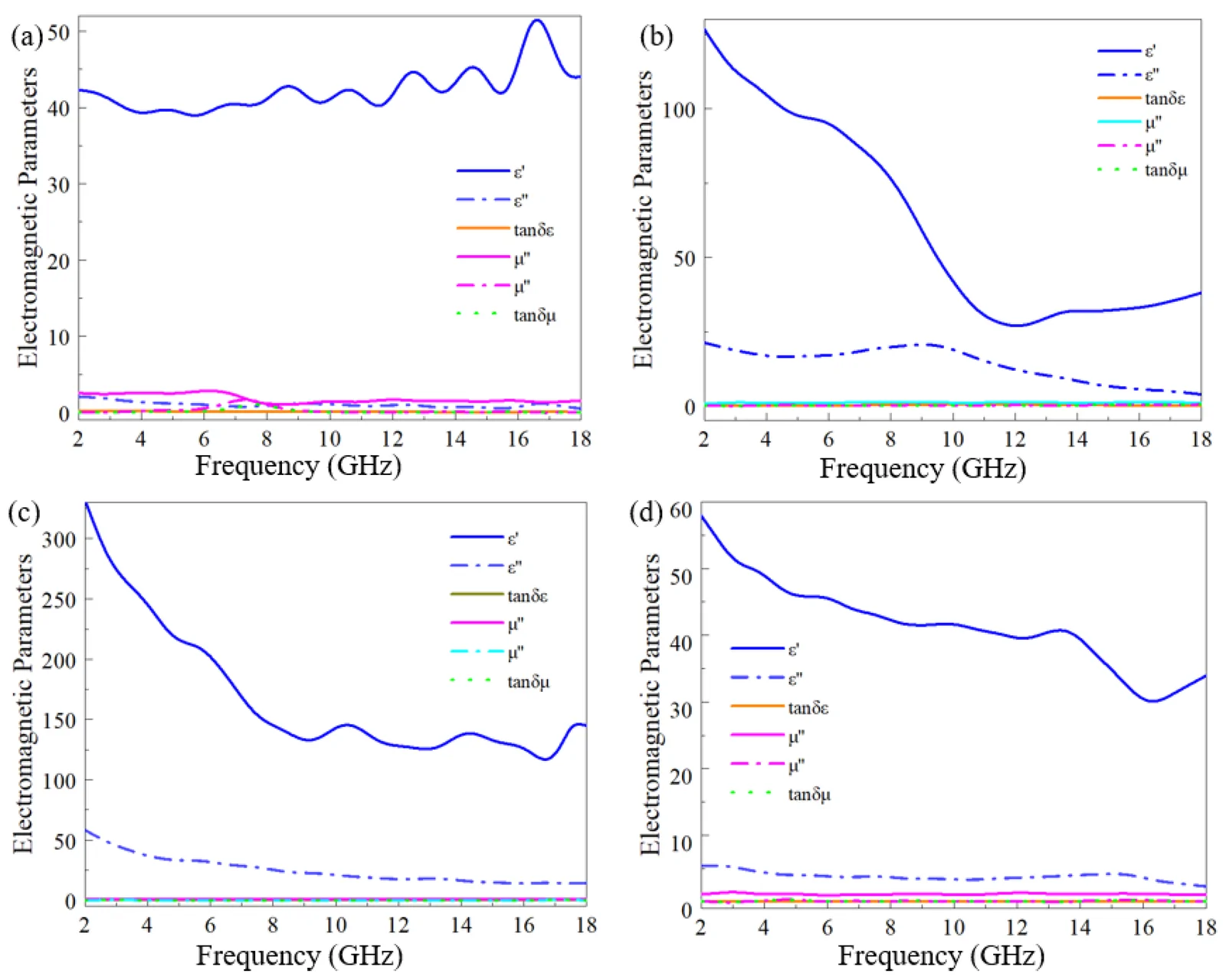
Electromagnetic parameters of (**a**–**d**) CKF/WPU-1 to CKF/WPU-4 measured in the range of 2–18 GHz.

The complex permittivity and permeability of CKF-1 to CKF-4 are shown in Figure 2. As the carbonization temperature rose from 600 to 1000 °C, the real and imaginary parts of the permittivity increased markedly. The highest permittivity of CKF-3 is attributed to the enhanced graphitization and carbon content at higher temperature, which raises electrical conductivity and strengthens electron conduction and dipole polarization. The large jump from CKF-2 (800 °C) to CKF-3 (1000 °C) corresponds to the steep rise in graphitization in this window: between 800 and 1000 °C, the residual functional groups are largely removed and the graphitic domains grow rapidly, so the carbon matrix crosses from a semiconducting to a conducting state [31]. The complex permeability stays close to unity for all samples because the material is non-magnetic carbon; the small μ″ fluctuation visible in Figure 3b reflects a weak residual response from trace impurities rather than an intentional magnetic-loss mechanism [32]. At 1200 °C, carbonization was accompanied by enlarged lattice defects and partial loss of the conductive network integrity, which lowered the permittivity. For filtering materials, a higher permittivity means a stronger response to electromagnetic waves and better frequency selectivity, so the CKF carbonized at 1000 °C shows the most promising filtering performance among all the CKF samples. The detailed characteristics are summarized in Table S3.

The reflection loss of CKF-1 to CKF-4 was calculated from the measured parameters (Figure S2 in the Supplementary Materials). The RL_min_ and the EAB were −11.3, −21, −14.7, and −12.1 dB and 6.8, 8, 1.1, and 1.4 GHz, respectively (Table S3). CKF-3, with the highest permittivity, shows the narrowest EAB (1.1 GHz), because a strong frequency-selective material reflects high-frequency microwaves and transmits low-frequency ones while absorbing very little. The poor absorption together with the strong frequency selectivity confirms that CKF-3 is the best wave-filtering material among all the CKF samples. Below 1000 °C, the rising carbonization temperature progressively removes oxygen- and hydrogen-containing functional groups and promotes ordering of the graphitic domains, so conductivity and polarization are enhanced [33]. Above 1000 °C, excessive thermal energy induces collapse of the thin fiber walls and agglomeration of the conductive domains, which breaks the continuity of the local conduction pathways and reduces the permittivity. The optimal temperature of 1000 °C therefore represents a balance between the conductivity gain and the structural integrity, and CKF-3 combines a high permittivity with a hollow microtube architecture that suits the frequency-selective filtering application.

Notably, the permittivity of the composite exceeds that of loosely packed bare CKF, a difference rooted in packing density. When bare CKF is pressed into a ring, its fiber-to-fiber contact is poor and the air-void content is high; since air has ε′ ≈ 1, it dilutes the effective permittivity strongly. At 20 wt% CKF, the polyurethane binder fills these voids, improves inter-tube contact, and replaces low-ε air (ε′ ≈ 1) with polyurethane (ε′ ≈ 3–5). The result is a denser, better-connected network. When the CKF loading rises further to 30–40 wt%, however, fibers agglomerate and new voids appear, which disrupt the network and pull the permittivity back down—a typical percolation peak at intermediate loading.

Because bare CKF is difficult to use directly in engineering applications, the best filtering CKF-3 was compounded with waterborne polyurethane at different mass ratios. The complex permittivity of CKF/WPU-1 to CKF/WPU-4 is shown in Figure 3. With increasing CKF content, the permittivity of the composite films rises, because more high-permittivity CKF favors the construction of an interconnected conductive network. At a WPU:CKF ratio of 8:2 (CKF/WPU-3), the conductive pathways are optimally built, and the permittivity reaches its maximum with a real part as high as about 325. An excessive amount of CKF introduces too many voids, weakens the continuity of the conductive network, and reduces the permittivity, as observed for CKF/WPU-1. The introduction of polyurethane modifies the microstructure and electrical property distribution of the CKF, allowing the formation of a three-dimensional conductive network under specific conditions. This network homogenizes the internal electric field, enables charges to move and accumulate more efficiently, and significantly enhances the permittivity and hence the filtering performance of the films [34].

The reflection loss of CKF/WPU-1 to CKF/WPU-4 is shown in Figure S3 (Supplementary Materials). The RL_min_ and EAB values were −29.9, −25, −32.9, −47.6 dB and 2.1, 1.6, 1.5, 4.5 GHz, respectively (Table S4). CKF/WPU-3 possesses the narrowest EAB (1.5 GHz), which means the incident microwaves are partly reflected and partly transmitted rather than absorbed. This strong frequency selectivity makes CKF/WPU-3 the most appropriate filtering material among the four films. The evolution of permittivity with the mass ratio follows the percolation-like behavior of the conductive network. At a low CKF content (9:1), the carbon microtubes are isolated by the polyurethane matrix and the conductive pathways are scarce, so the permittivity is moderate. As the CKF content rises to 8:2, the neighboring microtubes start to contact and overlap, and a connected three-dimensional conductive network is established throughout the film, giving the highest permittivity. When the content is further increased to 7:3 and 6:4, the excess fibers create voids and air gaps, the network is partly disrupted by poor dispersion, and the permittivity drops again [35]. The intermediate content (8:2) thus corresponds to the optimum network density, at which the dielectric response and the mechanical integrity of the film are balanced. Based on the permittivity and reflection loss analyses, CKF carbonized at 1000 °C with a WPU:CKF ratio of 8:2 was selected as the optimal filtering film, hereafter denoted as CKF/WPU.

### 2.3. Broadband Absorption of the Sandwich Structure: Before and After Inserting the Filtering Layer

The single CKF/WPU layer is a poor absorber in isolation: its strong impedance mismatch with free space reflects most incident waves rather than dissipating them. Inserting it as a frequency-selective interlayer between a high-frequency surface layer and a low-frequency bottom layer turns this mismatch into a bandwidth-broadening advantage.

To validate the wave-filtering function of CKF/WPU, the film was inserted as an interlayer between a high-frequency absorbing surface layer (0.9 mm CPP) and a low-frequency absorbing bottom layer (0.1 mm FNP), and the reflection loss of the resulting sandwich composite was simulated and measured [36]. The design principle is that the surface layer preferentially absorbs the high-frequency components of the incident wave, the bottom layer absorbs the residual low-frequency components, and the middle filtering layer is transparent to low-frequency waves while reflecting high-frequency waves back to the surface layer, so that the two absorbing layers work synergistically over a broad frequency range. The electromagnetic parameters of CKF/WPU-1 to CKF/WPU-4 were imported into CST MWS as the dispersive parameters of the interlayer, and the resulting sandwiches were denoted S1 to S4. The EAB and RL_min_ of S1 to S4 were 6.93, 7.08, 9.27, and 7.36 GHz and −57.8, −49.2, −54.8, and −56.6 dB, respectively (Figure S4 and Table S5).

**Figure 4:**
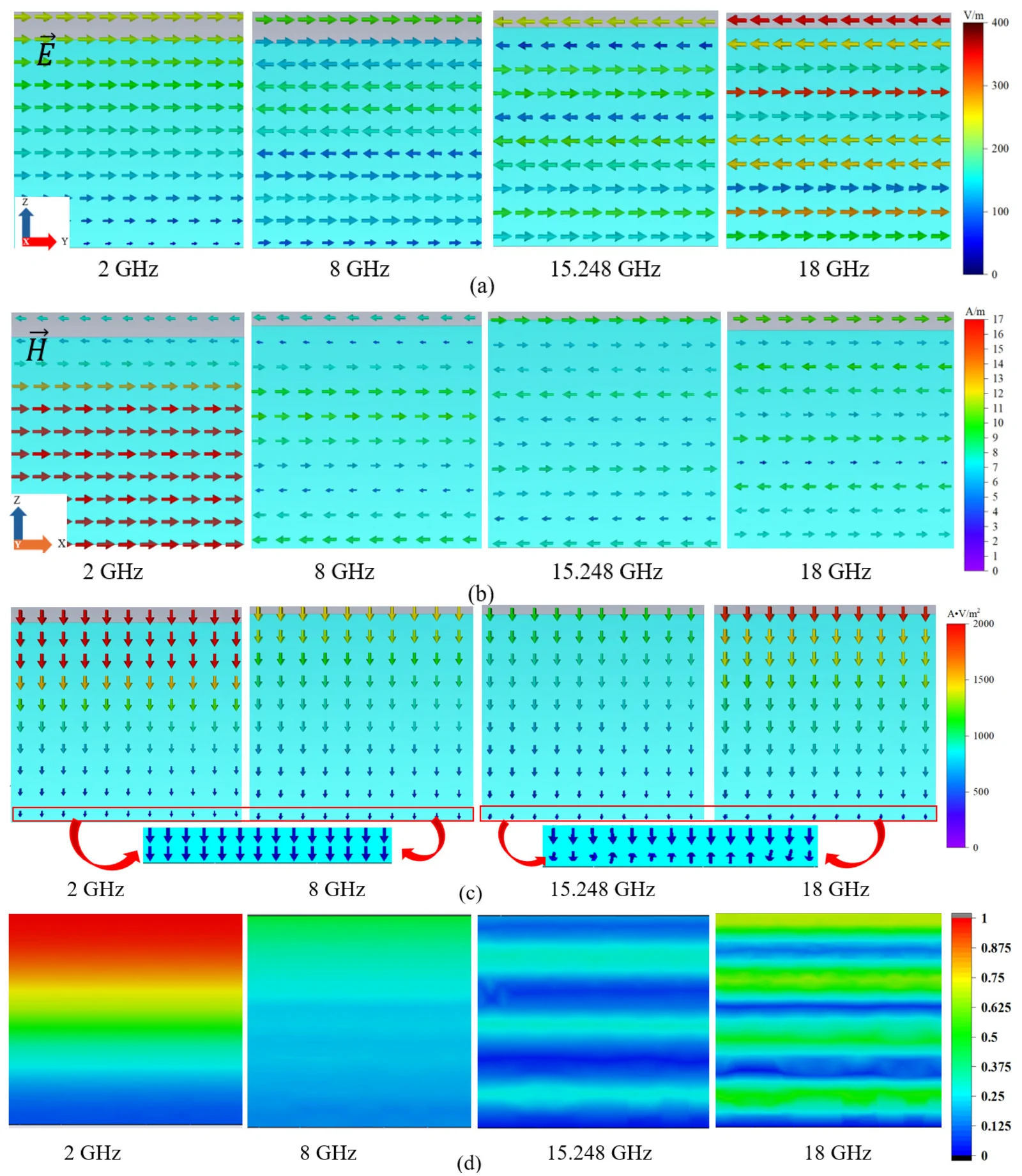
(**a**) Electric and (**b**) magnetic field vector distributions; (**c**) normalized power flow density and (**d**) power loss distributions of CKF/WPU at 2, 8, 15.25, and 18 GHz. The arrows indicate the directions of the electric and magnetic field vectors, and the color scale denotes the field amplitude.

The filtering function is supported by several independent observations. The single CKF/WPU layer itself is a poor absorber: its RL_min_ reaches only −9.12 dB at 0.62 mm and its EAB stays between 1.1 and 1.5 GHz (Table S4), so most incident energy is reflected or transmitted rather than dissipated. The CST field and power-flow maps (Figure 4) reveal the same behaviour from a different perspective: low-frequency waves pass through the film uniformly, whereas high-frequency ones pile up near the surface as the skin effect grows. The measured S3 sandwich completes the argument: its EAB of 9.27 GHz and RL_min_ of −54.8 dB is far beyond those of the two-layer reference without an interlayer (4.71 GHz, −43.6 dB; Table S5).

All four sandwiches show a wider EAB and a stronger RL_min_ than the two-layer composite without the interlayer (EAB = 4.71 GHz, RL_min_ = −43.6 dB). Among them, the insertion of the 0.62 mm CKF/WPU-3 interlayer gives the largest broadening of the effective absorption bandwidth. When the incident waves act on the high-permittivity CKF/WPU layer, a filtering effect occurs: the low-frequency microwaves pass through the layer and are absorbed by the bottom layer, while the high-frequency microwaves are reflected to the surface layer and absorbed there. This simultaneous low- and high-frequency absorption strengthens the absorption intensity and widens the bandwidth, confirming that the CKF/WPU film with a WPU:CKF ratio of 8:2 possesses the best filtering performance. The absorption dip between 9.3 and 11.6 GHz is not a shortcoming of the design but the transition region where neither the surface nor the bottom layer is impedance-matched and where the filtering interlayer itself merely transmits or reflects [37]. The benefit of this design, at only 1.53–1.62 mm total thickness, is coverage of the entire C band together with parts of the S and X bands—something a single-layer absorber of this thickness cannot achieve.

**Figure 5:**
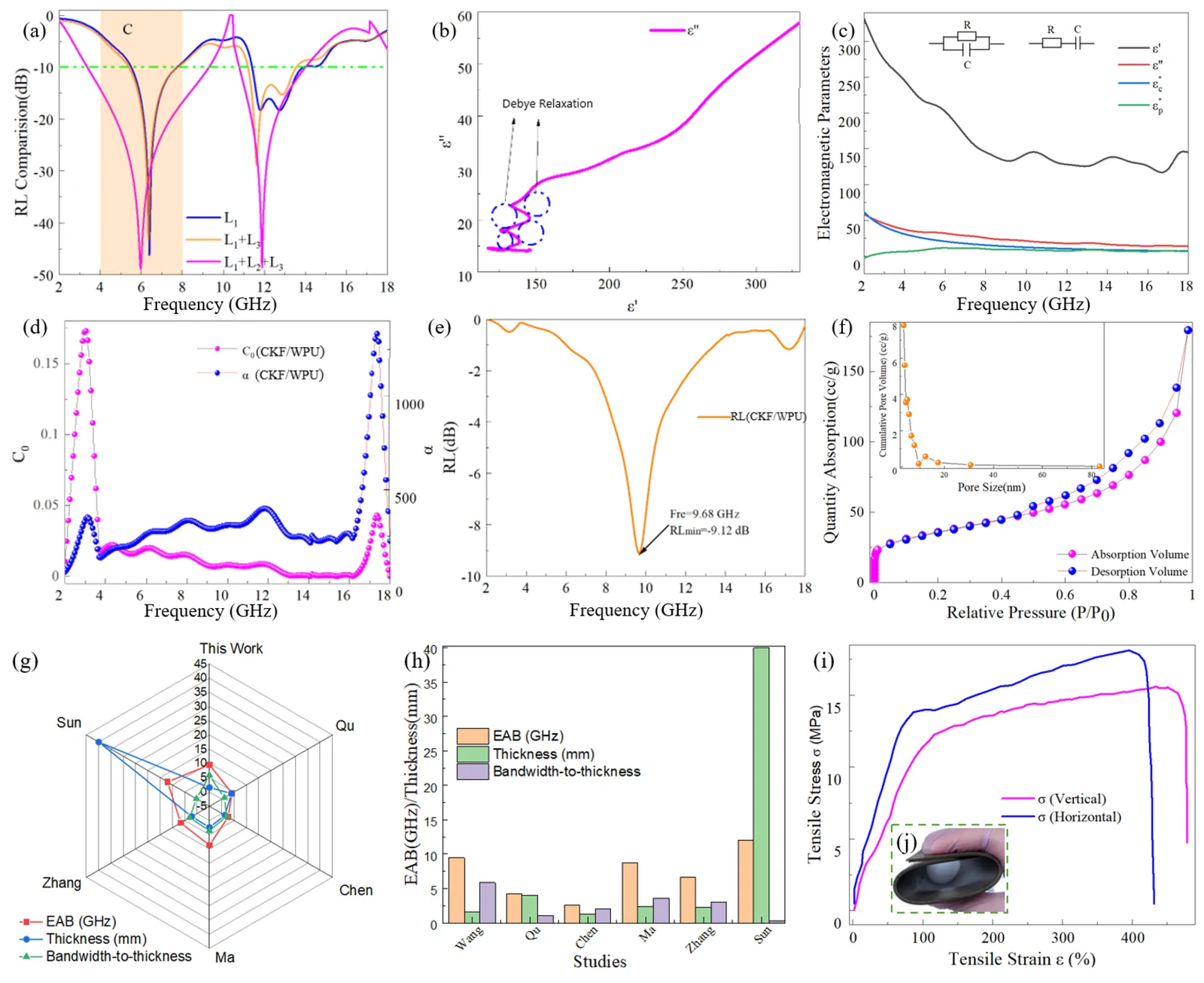
(**a**) RL comparison of composites; the orange shaded area denotes the C band (4–8 GHz). (**b**) Cole–Cole curve of CKF/WPU. (**c**) Equivalent-Circuit of CKF/WPU. (**d**) Magnetic resonance coefficient C_0_ (magenta) and attenuation constant α (blue) of CKF/WPU. (**e**) Reflection loss curve of the 0.62 mm CKF/WPU. (**f**) N_2_ adsorption–desorption isotherms of CKF. (**g**) Radar chart comparing the EAB, total thickness, and bandwidth-to-thickness ratio of this work with reported absorbers (Qu (2021) [43], Chen (2021) [44], Ma (2026) [45], Zhang (2026) [46], and Sun (2021) [47]). (**h**) EAB, total thickness, and bandwidth-to-thickness ratio of representative reported absorbers [43,44,45,46,47]. (**i**) Tensile stress–strain curves of the CKF/WPU film in the vertical and horizontal directions; the inset (**j**) is a digital photograph of the bent film, demonstrating its flexibility.

To verify the reliability of the simulated optimum thickness and the simulation model, a sandwich composite (S3) combining a 0.9 mm surface layer, a 0.62 mm CKF/WPU interlayer, and a 0.1 mm bottom layer was fabricated and measured with the VNA. The measured results show that at a total thickness of 1.53 mm, the RL_min_ of S3 is −54.8 dB at 5.97 GHz, and at a total thickness of 1.62 mm, the EAB is 9.27 GHz, given as two separated windows of 3.33–9.30 and 11.6–14.9 GHz, covering the whole C band together with parts of the S and X bands (Figure 5a). The simulation results agree well with the measured ones, confirming the reliability of the simulated thickness, the simplified model, and the condition settings, and demonstrating that the CKF/WPU interlayer can maximally broaden the effective absorption bandwidth of the composite at a thin total thickness. Compared with previously reported kapok-derived and carbon-textile absorbers, the sandwich composite with the CKF/WPU wave-filtering interlayer achieves both a broader EAB and a smaller total thickness, highlighting the advantage of the frequency-selective filtering design. The performance gain originates from the complementary spectral functions of the three layers. The high-frequency absorbing surface layer converts the high-frequency components of the incident wave into heat; the low-frequency absorbing bottom layer absorbs the low-frequency components that have passed through the interlayer; and the CKF/WPU interlayer acts as a frequency valve that prevents the high-frequency waves from reaching the bottom layer, where they would be poorly absorbed because of the impedance mismatch, while allowing the low-frequency waves to pass. In this way, the strong absorption of the two absorbing layers is extended over a much broader frequency range than either layer can cover alone, and the total thickness remains small because the thin filtering layer replaces the need for additional thick absorbing layers.

The electric and magnetic field vector distributions of the 0.62 mm CKF/WPU film at 2, 8, 15.25, and 18 GHz are shown in Figure 4a,b. At 2 GHz, the skin effect is weak, and the wave penetrates uniformly into the material, so both field vectors are uniformly distributed inside the film. As the frequency increases to 8 and 15.25 GHz, the skin effect becomes increasingly pronounced, and the field vectors are mainly concentrated near the surface. At 18 GHz the field energy exists only in an extremely thin surface layer, while the field strength inside the material is very weak. With increasing frequency, the periodic standing-wave field derived from the highly conductive metal backing is gradually enhanced, which reshapes the internal field distribution. Especially at 18 GHz, the capacitive structure formed between the metal backing and the rear surface of the material excites surface plasmons that significantly enhance the field strengths. Such a distribution favors the reflection of high-frequency microwaves but is unfavorable for overall absorption, which is consistent with the filtering behavior of the material [38].

According to the CST MWS simulation and Equations (1)–(4), the normalized power flow density and power loss distributions of CKF/WPU at the four frequencies are shown in Figure 4c,d. At 2 GHz, the permittivity is high and the conductivity is low, so the surface skin effect is weak; the wave can penetrate the material, making the power flow density uniformly distributed throughout the film. With increasing frequency, permittivity decreases and the skin effect is gradually enhanced, hindering penetration and shifting the power flow density toward the surface while weakening it inside the material. Especially at 15.25 and 18 GHz, the strong skin effect causes the incident waves to be reflected. The power loss distribution is consistent with this picture: at 2 GHz the weak skin effect leads to a uniform power loss under the action of the high permittivity, whereas at higher frequencies the skin effect and eddy current loss become concentrated near the surface, and the standing-wave energy of the metal backing enhances the loss ability especially at the surface and at the backing–film interface [39]. Accordingly, CKF/WPU favors the transmission of low-frequency microwaves and the reflection of high-frequency ones.

(1) $$\vec{S}=\vec{E}\times \vec{H}$$

(2) Sav→=1T∫0TS→dt=12Re[E→×H→]

(3) $$\delta =\sqrt{\frac{2}{\omega \mu \sigma }}=\frac{1}{\sqrt{\pi f\mu \sigma }}$$

(4) Ploss=−∇·Re[E→×H→]=−2∇·S→av

In summary, the microwave absorption loss of CKF/WPU mainly originates from the conduction loss and dielectric polarization loss of the CKF. The dielectric polarization loss comes from the dipole polarization of CKF, the abundant mesopores, and the discontinuous charge distribution provided by asymmetric interfaces such as the WPU/CKF heterogeneous interfaces and defects. A very small magnetic loss may arise from residual nitrogen-containing functional groups and trace ferromagnetic mineral residues that biomass-derived carbons commonly retain after high-temperature carbonization; the measured permeability remaining close to unity (Figure 2) and the absence of any crystalline magnetic phase in the XRD pattern (Figure 1b) both indicate that this contribution is minor compared with conduction and polarization loss. Although the single CKF/WPU layer possesses a large attenuation constant (α up to about 1700) that favors energy dissipation, the strong impedance mismatch with free space prevents most of the incident high-frequency waves from entering the material. As a result, the intrinsic loss capacity is not fully used in the single-layer configuration, and the material mainly acts as a frequency-selective reflector. This apparent contradiction between strong attenuation ability and weak absorption is the key feature of a wave-filtering material: it dissipates part of the wave energy while redistributing the rest, and its true value appears when it is combined with absorbing layers that have matched impedance.

### 2.4. Dielectric Loss and Frequency-Selective Filtering Mechanism

The Cole–Cole semicircle curves of CKF/WPU are shown in Figure 5b and Equations (5)–(7). Both curves are dominated by straight lines, indicating that the dielectric polarization mainly originates from conduction, while the Debye relaxation is weak. This is consistent with the high permittivity of CKF/WPU (ε′ ≈ 325), which reflects its well-connected conductive network. The fitted semicircles in the CKF/WPU curve are slightly more numerous than those of CKF, because the exposed mesopores of CKF increase the polarization at the heterogeneous interfaces after the addition of polyurethane. The dielectric polarization of CKF/WPU is therefore mainly attributed to the three-dimensional conductive channels constructed by the interwoven CKF, which enhance the conduction loss and are favorable to the absorption loss.

(5) $$\varepsilon \prime =\varepsilon_{\infty }+\frac{\varepsilon_{S}-\varepsilon_{\infty }}{1+{\left(2\pi f\right)}^{2}\tau^{2}}$$

(6) $$\varepsilon \prime \prime =\frac{2\pi f\tau (\varepsilon_{S}-\varepsilon_{\infty })}{1+{\left(2\pi f\right)}^{2}\tau^{2}}$$

(7) $$(\varepsilon \prime -\frac{\varepsilon_{S}+\varepsilon_{\infty }}{2})+{\left(\varepsilon \prime \prime \right)}^{2}={\left(\frac{\varepsilon_{S}-\varepsilon_{\infty }}{2}\right)}^{2}$$

To separate the conduction and polarization contributions, the measured permittivity was fitted with an equivalent-circuit model (ε″ = ε″c + ε″p; detailed decomposition, fitted conductivity σ ≈ 6.9 S/m, and representative values are given in Figure 5c and Table S2). The conduction term dominates at low frequency and decays as 1/f, while the polarization term remains small below ~6 GHz and rises slightly at high frequency, consistent with the Cole–Cole curves.

The magnetic resonance coefficient C_0_ (magenta) and the attenuation constant α (blue) of CKF/WPU are shown in Figure 5d and Equations (8) and (9). The C_0_ curves of the two materials show essentially the same trend with frequency, indicating that the magnetic loss ability of CKF/WPU mainly originates from the CKF. Both curves show a weak fluctuation around 3 GHz, which indicates a weak natural resonance that can be assigned to the residual nitrogen-containing functional groups and trace ferromagnetic mineral residues inherited from the raw kapok fibers, both being typical of biomass-derived carbons; since no magnetic phase was intentionally introduced and the permeability remains close to unity (Figure 2), this magnetic contribution is considered minor. In the 4–16 GHz range, the curves are nearly linear, indicating weak magnetic resonance. In the 16–18 GHz range, both curves again show large amplitudes, revealing eddy current loss. The attenuation constant α shows a consistent frequency dependence, with fluctuation peaks in the 2–4 and 16–18 GHz ranges and an almost linear variation in the 4–16 GHz range. The peak values of α for CKF/WPU and CKF are about 1700 and 1400, respectively; the larger value for CKF/WPU comes from the improved dispersion of CKF by the polyurethane, which increases the multiple reflection and scattering of the waves and enhances the attenuation ability.

(8) $$C_{0}=\frac{2\pi \sigma d^{2}}{3}=\mu \prime \prime {\left(\mu \prime \right)}^{-2}f^{-1}$$

(9) $$\alpha =\frac{\sqrt{2}\pi f}{c}\sqrt{(\mu \prime \prime \varepsilon \prime \prime -\mu \prime \varepsilon \prime )+\sqrt{{\left(\mu \prime \varepsilon \prime \prime +\mu \prime \prime \varepsilon \prime \right)}^{2}+{\left(\mu \prime \prime \varepsilon \prime -\mu \prime \varepsilon \prime \prime \right)}^{2}}}$$

The normalized input impedance of CKF/WPU is weak over most of the 2–18 GHz band: the narrow frequency window in which the film actually absorbs the incident energy is only about 1.1 GHz for the 0.62 mm sample (the full impedance-matching maps are provided in Figure S6 of the Supplementary Materials). Away from this window, most of the incident wave is either transmitted or reflected rather than dissipated, and this strong impedance mismatch with free space is precisely what gives the film its filtering character.

(I) The permittivity of the high-permittivity CKF/WPU is frequency-dependent (Figure 3c). With increasing frequency, the permittivity decreases: at low frequency, the permittivity is high, the propagation velocity of the wave in the material is low, and the wave passes through easily; at high frequency, the permittivity decreases, the propagation velocity increases, and reflection is enhanced.
(II) The skin effect of CKF/WPU is frequency-dependent. Low-frequency microwaves generate a weak skin effect with a large skin depth, so the long-wavelength low-frequency waves penetrate uniformly into the material and are transmitted. High-frequency microwaves generate a strong skin effect with a small skin depth, so the short-wavelength high-frequency waves are mostly reflected [40].
(III) The resonance characteristics of the high-permittivity material enable transmission or reflection at specific frequencies, producing a pronounced resonance peak. The RL curve of the 0.62 mm CKF/WPU film is shown in Figure 5e. At 9.68 GHz, the RL_min_ of the 0.62 mm CKF/WPU is only −9.12 dB, far from effective absorption, so the film only filters the waves, i.e., part of the waves is transmitted and another part is reflected. High-frequency microwaves cannot match the resonance frequency of CKF/WPU and are reflected, whereas mid- and low-frequency microwaves match the resonance frequency and are transmitted [41].
(IV) The mesopores tune the frequency selectivity. The N_2_ isotherm of CKF is type IV (Figure 5f), with a BET area of 112.98 m^2^/g. The pores form when inert gas escapes through the fiber wall during carbonization. After WPU compounding, the BET area drops sharply to 3.74 m^2^/g. The polymer fills most of the pores, so CKF/WPU shows only weak polarization loss, consistent with the small Cole–Cole semicircles in Figure 5b. The remaining pores behave differently at different wavelengths. For millimeter-scale high-frequency waves, they act as scattering centers and internal reflection interfaces, lengthening the propagation path and promoting reflection at the air–carbon boundary [42], as seen in the field and power-flow maps (Figure 4). For centimeter-scale low-frequency waves, the pores are far smaller than the wavelength and scatter almost nothing; the waves pass through the film and reach the bottom absorbing layer [41]. The moderate aspect ratio (≈29) helps as well: it extends the low-frequency path without over-attenuating the wave before it arrives. The WPU matrix also renders the film flexible and conformable to curved surfaces. As shown in Figure 5j, the 0.62 mm CKF/WPU film can be rolled and sharply bent without cracking, and the tensile stress–strain curves in Figure 5i quantify this compliance: the film delivers a tensile strength of 15–19 MPa together with an elongation at break above 400%, and the somewhat higher strength in the horizontal direction reflects the preferential in-plane alignment of the CKF introduced during stirring and hot pressing. Such mechanical compliance, rarely achieved in high-permittivity carbon films, allows the filtering layer to fit non-planar substrates. Finally, Figure 5g,h and Table S6 benchmark the sandwich against reported kapok-derived and carbon-based absorbers in terms of EAB, total thickness, and the bandwidth-to-thickness ratio: with an EAB of 9.27 GHz at a total thickness of only 1.53–1.62 mm, the present sandwich achieves one of the highest bandwidth-to-thickness ratios among the compared works [43,44,45,46,47].

**Figure 6:**
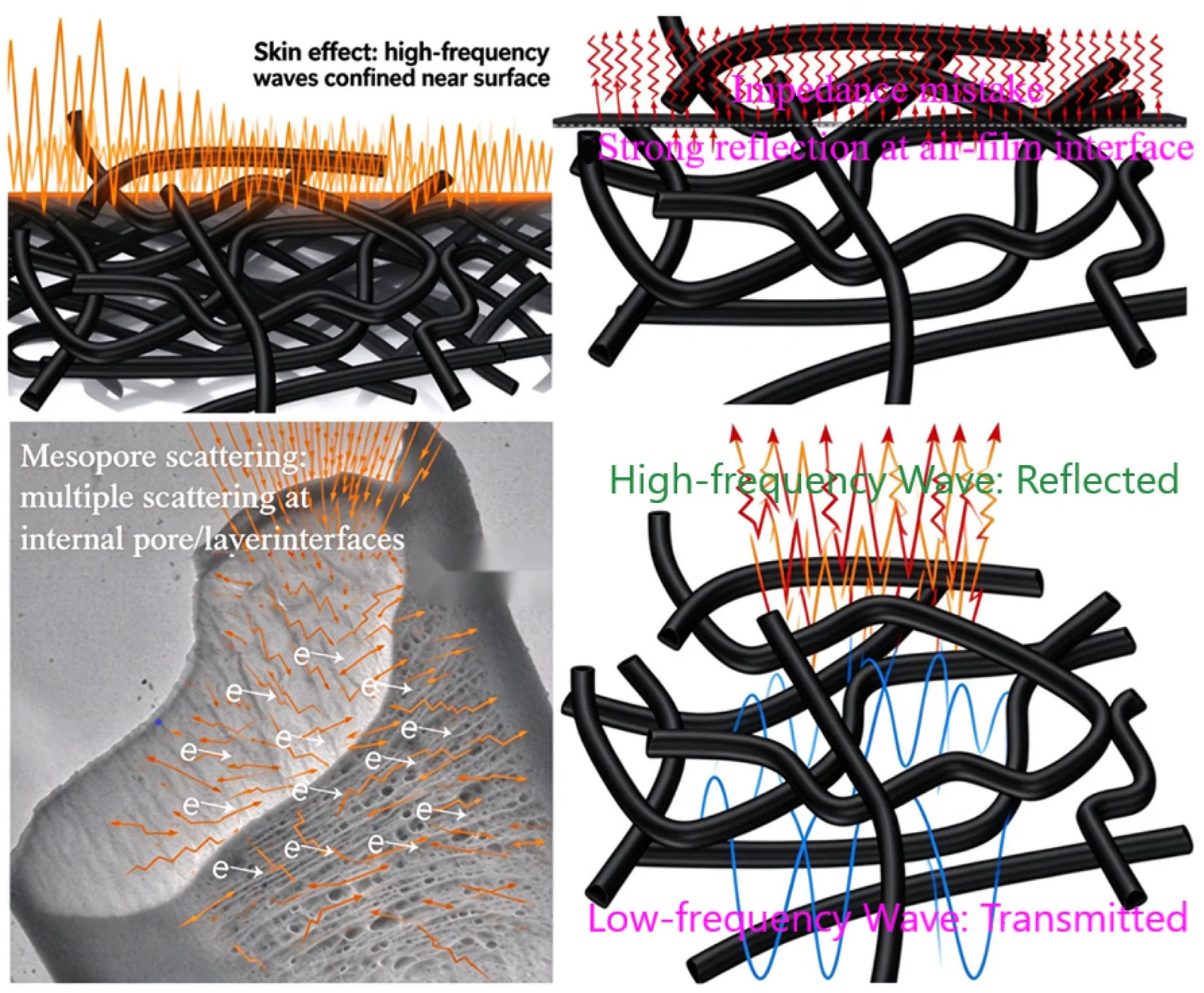
Schematic diagram of the microwave-filtering mechanism of CKF/WPU.

Quantitatively, the effective impedance-matching bandwidth remains below about 1.2 GHz across the practical thickness range of 0.43–0.62 mm (Figure S6), confirming the very weak impedance match with free space [48]. Figure 6 (with an extended schematic in Figure S5) schematically summarizes the proposed frequency-selective filtering mechanism of CKF/WPU, in which the frequency-dependent permittivity, the skin effect, the narrow resonance window, mesopore scattering, and the poor impedance match with free space jointly produce low-frequency transmission and high-frequency reflection.

## 3. Materials and Methods

### 3.1. Materials

Raw kapok fibers (Guangxi Zhaojie Company, Guangxi, Baise, China) were used as the carbon precursor. Sodium hydroxide (NaOH, analytical grade), anhydrous ethanol, and deionized water were purchased from Shanghai Macklin Biochemical Co., Ltd. (Shanghai, China). Waterborne polyurethane (PU-2540, solid content 40 wt%) was obtained from Guangzhou Yuheng Company (Guangzhou, China). All reagents were used as received.

### 3.2. Preparation of Carbonized Kapok Fibers

Kapok fibers were purified in an 8 g/L NaOH solution (ethanol: water = 2:8 by volume) at 95 °C for 1 h, washed to neutrality, and dried. The purified fibers were carbonized in a tube furnace under argon at 600–1200 °C (heating rate 5 °C/min, holding time 2 h), giving CKF-1 to CKF-4. The four carbonization temperatures were assigned as 600, 800, 1000, and 1200 °C for CKF-1, CKF-2, CKF-3, and CKF-4, respectively. This interval was chosen to span the transition from incomplete carbonization, in which oxygen- and hydrogen-containing groups remain and the conductivity is low, to excessive graphitization accompanied by partial collapse of the hollow fiber walls, so that the balance between conductivity gain and structural integrity could be identified experimentally.

### 3.3. Preparation of CKF/WPU Composite Films

**Figure 7:**
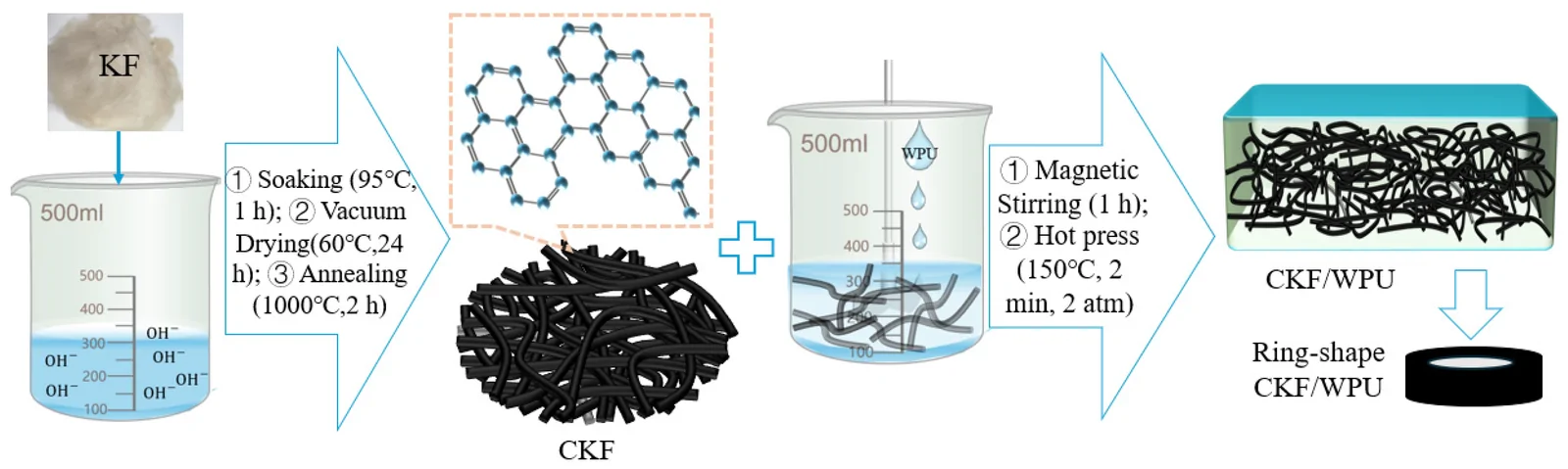
Schematic diagram of the main preparation process of CKF/WPU composite films.

The as-prepared CKF was dispersed in a diluted waterborne polyurethane solution under mechanical stirring, and CKF/WPU composite films were fabricated by hot pressing at 150 °C under a pressure of 2 atm for 2 min. The mass ratios of WPU to CKF were set to 6:4, 7:3, 8:2, and 9:1, and the composites were denoted CKF/WPU-1, CKF/WPU-2, CKF/WPU-3, and CKF/WPU-4, respectively. These four ratios were selected to scan the percolation regime of the conductive network: at low CKF loading, the carbon microtubes are isolated by the polyurethane matrix; near the optimum loading, they contact and overlap into a connected three-dimensional network; and beyond it, agglomeration and void formation degrade the network again. The films were cut into ring-shaped samples with inner and outer diameters of 3.04 and 7 mm for electromagnetic parameter measurement. Figure 7 shows the main preparation route.

### 3.4. Characterization

FTIR spectra were recorded on a Nicolet iS50 spectrometer (Thermo Fisher Scientific, Waltham, MA, USA; 4000–400 cm^−1^). SEM and TEM images were taken with a FlexSEM1000 (Hitachi, Tokyo, Japan) and an H7650 (Hitachi, Tokyo, Japan). XRD patterns were collected on a D8 ADVANCE (Bruker AXS, Karlsruhe, Germany). N_2_ adsorption–desorption isotherms were measured with an Autosorb-iQ analyzer (Quantachrome Instruments, Boynton Beach, FL, USA), and the specific surface area was calculated by the BET method. Raman spectra were collected with a Renishaw spectrometer (Renishaw, Wotton-under-Edge, UK).

### 3.5. Electromagnetic Parameter and Reflection Loss Measurement

The complex permittivity (ε_r_ = ε′ − jε″) and complex permeability (μ_r_ = μ′ − jμ″) of the ring-shaped samples were measured with an E5071C vector network analyzer (VNA, Agilent Technologies, Santa Clara, CA, USA) by the coaxial transmission line method over 2–18 GHz with 201 scanning points. The RL of the samples with metal backing was calculated from the measured parameters based on transmission line theory, and the quarter-wavelength matching condition was used as the theoretical reference for the thickness dependence of the absorption peaks, as shown in Equations (10)–(12) and (13), respectively.

(10) $$Z_{in}=Z_{0}\sqrt{\frac{\mu_{r}}{\varepsilon_{r}}}\left[j\left(\frac{2\pi fd}{c}\sqrt{\varepsilon_{r}\mu_{r}}\right)\right]$$

(11) $$RL=20\log|\frac{Z_{in}-1}{Z_{in}+1}|$$

(12) $$Z_{0}=\sqrt{\frac{\mu_{0}}{\varepsilon_{0}}}$$

(13) $$d=\frac{nc}{4f\sqrt{\mu_{r}\varepsilon_{r}}}\;(n=1,3,5,\ldots )$$

### 3.6. Simulation

Electromagnetic simulations were carried out with CST Microwave Studio (2025). The single-layer and sandwich models used the measured dispersive permittivity; full boundary conditions, mesh settings, and layer geometries are given in Section S7.

## 4. Conclusions

Carbonized kapok fibers (CKFs) and CKF/waterborne polyurethane composite films were fabricated by high-temperature carbonization and hot pressing. The CKF obtained at 1000 °C, with a hollow microtube architecture and moderate graphitization (I_D_/I_G_ ≈ 0.73), showed the highest permittivity and strongest frequency-selective behavior. Compounding it with WPU at a mass ratio of 8:2 raised the dielectric real part to about 325 via an optimally built three-dimensional conductive network, yielding an efficient wave-filtering layer that transmits low-frequency microwaves while reflecting high-frequency ones. The filtering effect originates from the frequency-dependent permittivity and skin effect, a narrow resonance window, abundant mesopores, and the poor impedance match with free space. Inserting a 0.62 mm CKF/WPU interlayer broadened the effective absorption bandwidth from 4.71 to 9.27 GHz (3.33–9.30 and 11.6–14.9 GHz) with an RL_min_ of −54.8 dB at only 1.53–1.62 mm total thickness, covering the whole C band. The approach is expected to extend to other biomass-derived hollow carbons as flexible filtering interlayers. Owing to the flexibility and low areal density imparted by the WPU matrix—the film sustains a tensile strength of 15–19 MPa at an elongation at break above 400% and can be rolled and bent without cracking (Figure 5i,j)—it can conform to curved and moving surfaces, such as drone wings, aircraft leading edges, and protective garments, where thick magnetic or rigid ceramic absorbers are difficult to deploy; bending-cycle fatigue tests will be reported in follow-up work.

## Data Availability

The raw data supporting the conclusions of this article will be made available by the authors on request.

## Supplementary Materials

The following supporting information can be downloaded at: https://www.mdpi.com/article/10.3390/molecules31213985/s1, Section S1. Experimental Details. Section S2. Raman Characterization of CKF. Section S3. Equivalent-Circuit Decomposition of the Dielectric Loss. Section S4. Quarter-Wavelength Resonance and Skin-Depth Estimates. Section S5. Reflection Loss of the Individual Layers. Section S6. Reflection Loss of the Sandwich Composites. Section S7. Simulation Settings. Section S8. Normalized Impedance Matching Maps. Section S9. Comparison of microwave absorption of samples. Figure S1. (a,b) Diameter and length distribution of CKF. (c) Fiber wall thickness distribution of CKF. Figure S2. (a,c,e,g) Color maps and (b,d,f,h) contour plots of the reflection loss of CKF-1 to CKF-4. Figure S3. (a,c,e,g) Color maps and (b,d,f,h) contour plots of the reflection loss of CKF/WPU-1 to CKF/WPU-4. Figure S4. (a,c,e,g) Color maps and (b,d,f,h) contour plots of the reflection loss of the sandwich composites combined with CKF/WPU-1 to CKF/WPU-4 as the interlayer. Figure S5. Schematic diagram of the microwave-absorbing mechanism of CKF/WPU. Figure S6. Normalized input impedance (Z_in_/Z_0_) of the CKF/WPU film. Table S1. Peak parameters of the Raman spectrum of CKF-3. Table S2. Representative values of the equivalent-circuit decomposition. Table S3. Electromagnetic and microwave absorption characteristics of the carbonized kapok fibers (CKFs) prepared at different carbonization temperatures. Table S4. Electromagnetic and microwave absorption characteristics of the CKF/WPU composite films with different mass ratios. Table S5. Microwave absorption performance of the sandwich composites with different CKF/WPU interlayer thicknesses. Table S6. Comparison of microwave absorption of samples.
