# Supplementary material for: High-Permittivity Carbonized Kapok Fiber/Waterborne Polyurethane Composite Films as Frequency-Selective Filtering Layers for Thin-Profile Multiband Microwave Absorption
Source: Molecules. 2026 Nov 5;31(21):3985. doi: 10.3390/molecules31213985 (PMC13765054; doi:10.3390/molecules31213985)
Supplement: Supplementary file 1 [file molecules-31-03985-s001.zip › molecules-4660256-supplementary.pdf]

Supporting Information

Section S1 Experimental Details

The experimental details of CPP and FNP were shown in https://doi.org/10.1016/j.cej.2026.176008.

Section S2 Raman Characterization of CKF

The Raman spectrum of CKF-3 was recorded at three positions (files 1-1, 1-2, and 1-3), and the three traces nearly overlap, indicating that the carbonized fibers are uniform. Two bands are identified: the D band at 1334.26 cm^{-1} and the G band at 1593.40 cm^{-1}. The peak-height ratio ID/IG is 1546.04/2130.14 ≈ 0.73, and the peak parameters are listed in Table S1.

Table S1. Peak parameters of the Raman spectrum of CKF-3 (from the peak-picking table of the WiRE software).

Peak	Centre (cm^{-1})	Height (counts)	Width (cm^{-1})	Area (a.u.)
1 (D band)	1334.26	1546.04	87.84	151372
2 (G band)	1593.40	2130.14	65.77	175593

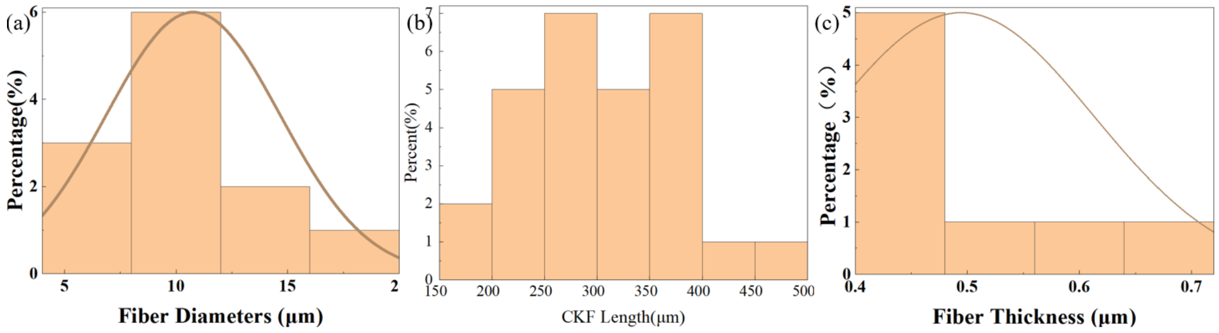

Figure S1. (a,b) Diameter and length distribution of CKF. (c) Fiber wall thickness distribution of CKF.

Section S3 Equivalent-Circuit Decomposition of the Dielectric Loss

To separate the conduction and polarization contributions, the measured permittivity was fitted with an equivalent-circuit model. The imaginary part of the permittivity is written as

$$\varepsilon''(f) = \varepsilon''c(f) + \varepsilon''p(f), \quad \varepsilon''c = \sigma/(2\pi f\varepsilon_0)$$

where $\varepsilon''c$ is the conduction (Ohmic) contribution governed by the DC conductivity σ of the conductive network, and $\varepsilon''p$ collects the polarization relaxation contributions (Debye-type relaxation and interfacial polarization). Figure S2 plots ε′, ε″, $\varepsilon''c$, and $\varepsilon''p$ against frequency, and Table S2 lists the representative values. Two features are worth noting. First, $\varepsilon''c$ follows a clean 1/f decay (from about 61.7 at 2 GHz to about 6.9 at 18 GHz), which is exactly the

frequency dependence expected for a conductive network; the corresponding DC conductivity is nearly constant at σ ≈ 6.9 S/m over the whole band. Second, ε″p stays near zero at low frequency, rises to about 11 around 6 GHz, and remains around 7–9 at high frequency, so the two contributions become comparable above about 10 GHz. This indicates that conduction loss is the dominant dielectric-loss channel and interfacial polarization assists it at high frequency, consistent with the Cole–Cole curves in the main text (Figure 8(b)), which are dominated by straight lines rather than semicircles.

Note: the decomposition was carried out on the measured complex permittivity of CKF/WPU-3 (201 points over 2–18 GHz). The fitted permeability stays close to unity (μ′ = 0.73–1.20, μ″ < 0.50), as expected for a purely dielectric carbon film.

Table S2. Representative values of the equivalent-circuit decomposition (full data: 201 points, 2–18 GHz, available from the authors).

Frequency (GHz)	ε′	ε″	ε″c	ε″p
2.0	332.3	58.44	61.69	−3.25
2.5	298.8	51.37	49.36	2.01
3.0	274.5	45.60	41.14	4.46
3.5	259.5	40.98	35.26	5.73
4.0	245.2	37.15	30.85	6.30
4.5	228.1	34.57	27.42	7.15
5.0	216.1	33.45	24.68	8.77
5.5	211.2	33.07	22.43	10.64
6.0	202.0	31.93	20.56	11.37
6.5	186.4	29.98	18.98	11.00
7.0	168.6	28.65	17.63	11.03
7.5	153.7	27.41	16.45	10.96
8.0	145.2	25.43	15.42	10.01
8.5	138.6	23.70	14.52	9.19
9.0	133.3	22.90	13.71	9.19
9.5	135.3	22.32	12.99	9.33
10.0	142.9	21.24	12.34	8.90
10.5	145.0	20.01	11.75	8.26
11.0	138.2	19.13	11.22	7.91
11.5	130.9	18.30	10.73	7.57
12.0	128.1	17.70	10.28	7.41
12.5	126.5	17.79	9.87	7.92
13.0	125.7	18.16	9.49	8.66
13.5	130.3	17.78	9.14	8.64
14.0	137.2	16.53	8.81	7.71
14.5	137.7	15.47	8.51	6.96
15.0	132.8	14.98	8.23	6.75

Frequency (GHz)	ε′	ε″	ε″c	ε″p
15.5	129.7	14.46	7.96	6.50
16.0	125.8	14.19	7.71	6.48
16.5	118.2	14.48	7.48	7.00
17.0	121.8	14.53	7.26	7.27
17.5	142.8	14.11	7.05	7.06
18.0	145.1	14.33	6.85	7.47

Section S4 Quarter-Wavelength Resonance and Skin-Depth Estimates

Section S4.1 Quarter-wavelength condition

The 0.62 mm CKF/WPU film shows a reflection-loss dip of only −9.12 dB at 9.68 GHz. The quarter-wavelength condition $d = \lambda_0/(4\sqrt{\varepsilon' r})$, with λ_0 = 30.97 mm at 9.68 GHz, gives an effective permittivity of $\varepsilon'_{eff} = (30.97/(4 \times 0.62))^2 \approx 156$ for d = 0.62 mm. This value lies inside the measured range of ε′ (125–325) at that frequency, so the dip is consistent with a quarter-wave resonance of the thin film. At resonance, the film is partially transparent; away from it, the waves are increasingly reflected.

Section S4.2 Skin depth

The DC conductivity estimated from the conduction contribution of the dielectric loss is $\sigma \approx 6.9$ S/m (Section S3). For a lossy medium the skin depth is $\delta = \sqrt{2/(\omega\mu\sigma)}$. Using this estimate gives $\delta \approx 4.3$ mm at 2 GHz and $\delta \approx 1.4$ mm at 18 GHz. The skin depth therefore decreases by a factor of about three across the measured band and is comparable to or larger than the film thickness at low frequency. In a material with a permittivity as high as that of CKF/WPU, the penetration is further suppressed by the strong impedance mismatch at the air–film interface, and the CST field distributions (Figures 8 and 9 of the main text) directly show that the field energy shifts toward the film surface as the frequency rises, which is the physical basis of the low-frequency transmission and high-frequency reflection.

Section S5 Reflection Loss of the Individual Layers

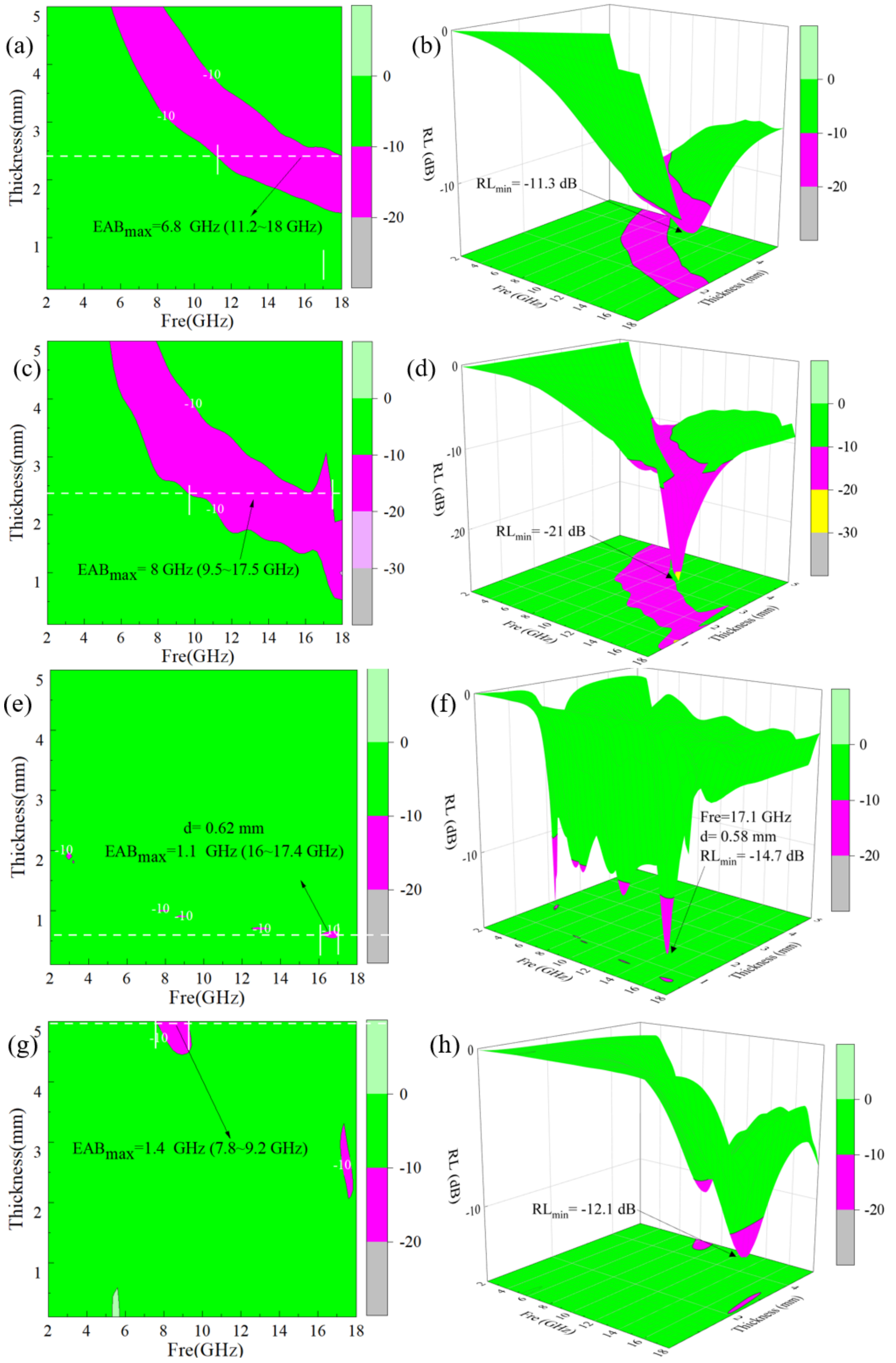

Figure S2. (a, c, e, g) Color maps and (b, d, f, h) contour plots of the reflection loss of CKF-1 to CKF-4.

Table S3. Electromagnetic and microwave absorption characteristics of the carbonized kapok fibers (CKFs) prepared at different carbonization temperatures.

Sample	Carbonization temperature (°C)	Permittivity ($\varepsilon' - j\varepsilon''$)	RL_{min} (dB)	EAB (GHz)
CKF-1	600	2.8 – j*1.5	−11.3	6.8
CKF-2	800	3 – j*0.2	−21	8
CKF-3	1000	(60–150) – j*(20–60)	−14.7	1.1
CKF-4	1200	(10–120) – j*(0–40)	−12.1	1.4

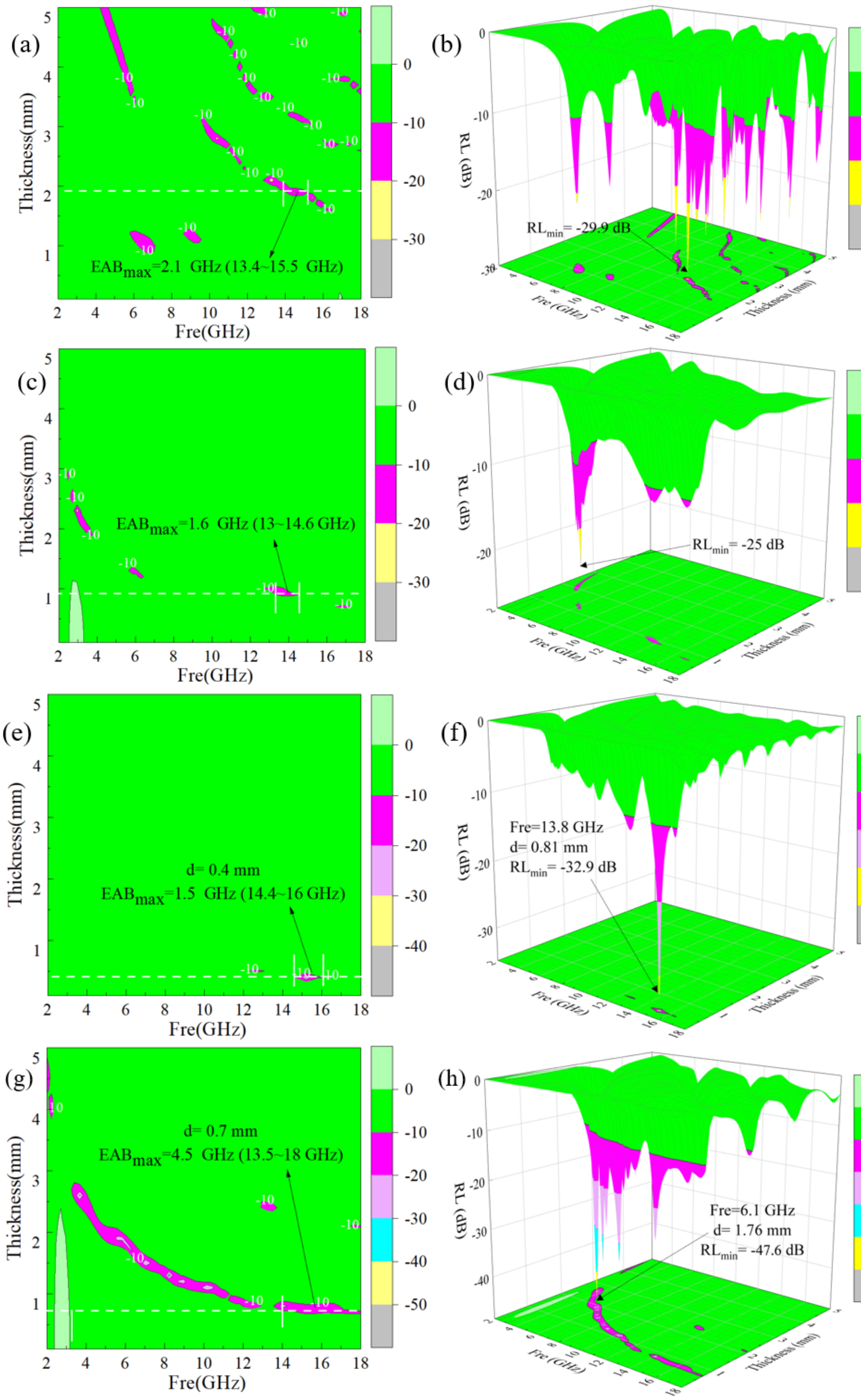

Figure S3. (a, c, e, g) Color maps and (b, d, f, h) contour plots of the reflection loss of CKF/WPU-1 to CKF/WPU-4.

Table S4. Electromagnetic and microwave absorption characteristics of the CKF/WPU composite films with different mass ratios.

Sample	WPU: CKF mass ratio	Permittivity ($\varepsilon' - j\varepsilon''$)	RL_{min} (dB)	EAB (GHz)
CKF/WPU-1	6:4	45 – j*2	−29.9	2.1
CKF/WPU-2	7:3	(25–125) – j*20	−25	1.6
CKF/WPU-3	8:2	(125–325) – j*(2–60)	−32.9	1.5
CKF/WPU-4	9:1	50 – j*5	−47.6	4.5

Section S6 Reflection Loss of the Sandwich Composites

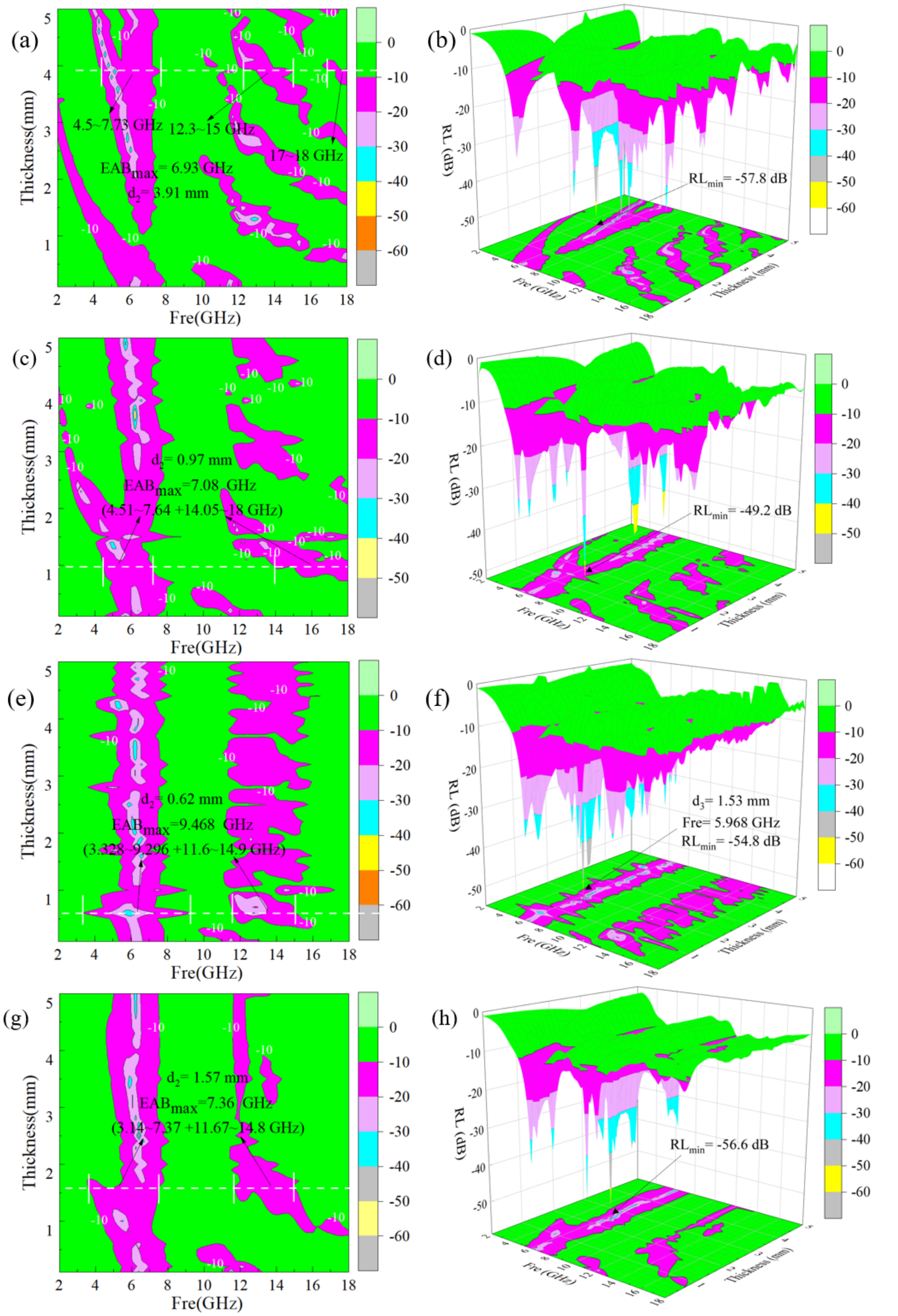

Figure S4. (a, c, e, g) Color maps and (b, d, f, h) contour plots of the reflection loss of the sandwich composites combined with CKF/WPU-1 to CKF/WPU-4 as the interlayer.

Table S5. Microwave absorption performance of the sandwich composites with different CKF/WPU interlayer thicknesses.

Composite	Interlayer	Total thickness (mm)	RL_{min} (dB)	EAB (GHz)
Two-layer reference	None	–	−43.6	4.71
S1	CKF/WPU-1	–	−57.8	6.93
S2	CKF/WPU-2	–	−49.2	7.08
S3	CKF/WPU-3	1.53–1.62	−54.8	9.47
S4	CKF/WPU-4	–	−56.6	7.36

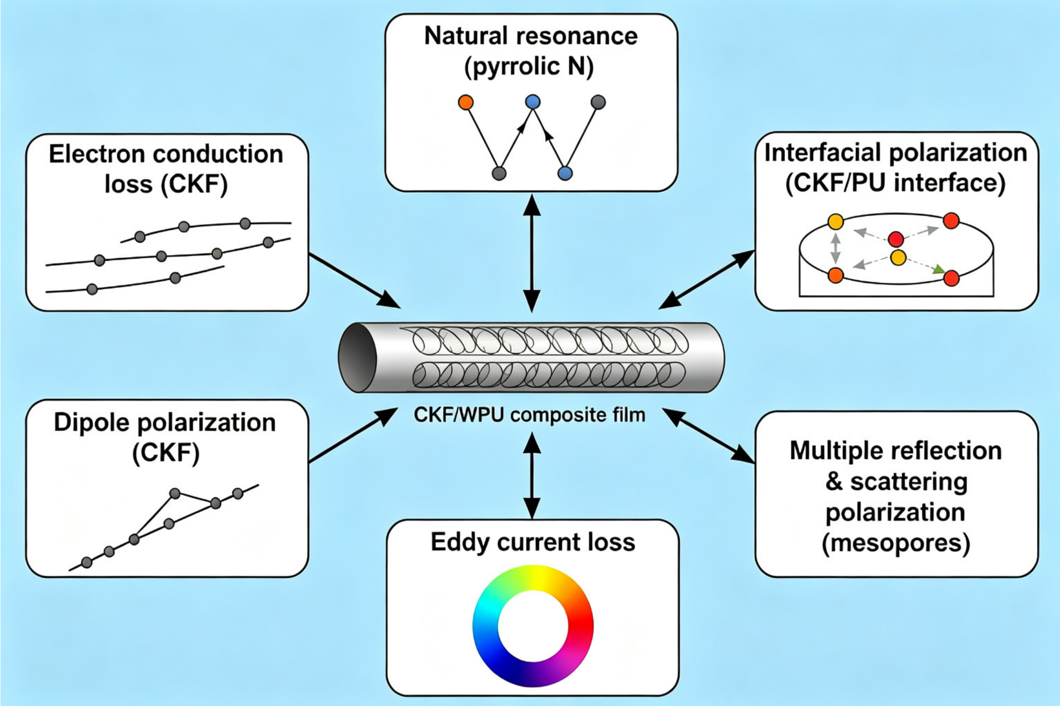

Figure S5. Schematic diagram of the microwave-absorbing mechanism of CKF/WPU.

Section S7 Simulation Settings

All simulations were performed with CST Microwave Studio 2025 (Dassault Systèmes). The single-layer model used the measured dispersive permittivity of the corresponding film. The sandwich model combined a 0.9 mm PCF/WPU surface layer, the CKF/WPU interlayer (0.43–0.62 mm sweep), and a 0.1 mm NFP bottom layer. The frequency-domain solver was used with open boundary conditions, and the parameter sweep and optimization functions were applied to determine the optimum interlayer thickness. The simulated reflection loss, electric and magnetic field vector distributions, normalized power flow density, and power loss were extracted for mechanism analysis. The same simulation settings were used for all samples, so the comparison between samples and between simulation and experiment is internally consistent.

Section S8 Normalized Impedance Matching Maps

The normalized input impedance (Zin/Z0) of the single CKF/WPU film was mapped as a function of frequency (2–18 GHz) and thickness (0.3–2.0 mm) from the measured complex permittivity and transmission-line theory. Figure S7 shows that the frequency range in which |Zin/Z0| lies close to unity — i.e., the film actually admits and absorbs the incident wave — is extremely narrow: about 1.1 GHz for the 0.62 mm sample, and only 0.9 and 1.2 GHz at thicknesses of 0.43 and 0.52 mm, respectively. Away from this narrow window, |Zin/Z0| deviates strongly from unity, so most of the incident energy is reflected at the air–film interface and cannot enter the material. Quantitatively, this confirms that CKF/WPU is a poor single-layer absorber over most of the band and acts as a frequency-selective filter that transmits low-frequency waves to the bottom absorbing layer while reflecting high-frequency waves back to the surface layer, consistent with the sandwich design in the main text.

Figure S7. Normalized input impedance (Z_{in}/Z_0) of the CKF/WPU film as a function of frequency (2–18 GHz) and thickness (0.3–2.0 mm). The narrow band where $|Z_{in}/Z_0| \approx 1$ corresponds to weak absorption; the rest of the band is dominated by reflection/transmission, i.e., filtering behavior.

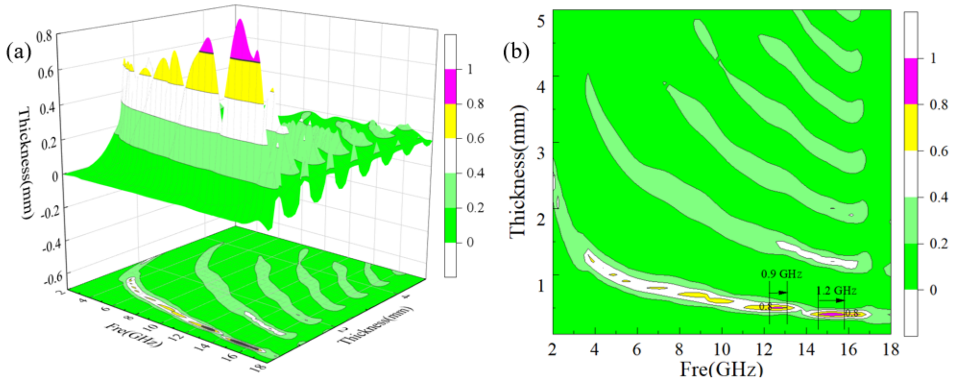

Figure S6. Normalized input impedance (Z_{in}/Z_0) of the CKF/WPU film.

Section S9. Comparison of microwave absorption of samples

Table S6 Comparison of microwave absorption of samples

Authors	Samples	Thickness (mm)	EAB (GHz)	Bandwidth-to-thickness
Wang	This work	1.62	9.47	5.84568
Qu [43]	FeCoNiOx-rGO/WPU/PI	3.97	4.2	1.05793
Chen [44]	CF@Co-ZIF	1.3	2.6	2
Ma [45]	Zn-benzimidazole	2.41	8.72	3.61826
Zhang [46]	bamboo-derived porous carbon (BPC)	2.2	6.65	3.02273
Sun [47]	CB-PLA metastructures	40	12	0.3
